# Announcement

**Published:** 1876-06

**Authors:** 


### *Announcement.*

Doctor E. H. Gibbs, the surviving partner of the late Dr. W. W. Hall, will continue the practice of medicine at the old office, No. 137 Eighth Street, near Broadway. All letters should be addressed as above.

